# American Medical Association

**Published:** 1892-07

**Authors:** 


### AMERICAN MEDICAL ASSOCIATION.

### FORTY-THIRD ANNUAL MEETING, HELD IN DETROIT, MICH., JUNE 7TH, 8TH, 9TH AND 10TH, 1892.

### FIRTT DAY, TUESDAY, JUNE 7TH—GENERAL SESSION.

The forty-third annual meeting of the American Medical Association was held in the Detroit Opera House on the Campus Martius. The meeting was called to order at 11 o'clock by Dr. H. O. Walker, chairman of the committee of arrangements.

After prayer by the Rt. Rev. Thos. F. Davis, Episcopal Bishop of Michigan, a brief address of welcome was delivered by General R. A. Alger. The speaker expressed the pleasure he felt in welcoming to Detroit and the State of Michigan so distinguished a body. The people would do all in their power to make their sojourn a pleasant one, and every door was open to whoever sought admittance. If the medical guest did not see what he wanted, he had only to send for it; and if he did see what he wanted, he must help himself to it.

Dr. H. O. Walker supplemented this address by a few words of cordial welcome from the profession of the city, whose honor it was to entertain for the third time the members of the American Medical Association. He then introduced the president of the association, Dr. Henry O. Marcy, of Boston, presenting him with a handsome gavel made from the wood of the celebrated Pontiac tree, a tree which was a silent witness of the final triumph of the infant settlement of Detroit over its savage foes.

The president thanked the committee for its gift, and said that he felt a peculiar pleasure in being able to reciprocate by presenting to the Association a gavel with no less precious historic associations. It was made from a piece of the Charter Oak of Hartford, with a handle of wood from the United States frigate "Constitution."

### "EVOLUTION IN MEDICINE"

was the subject of the President's annual address. The American Medical Association, he said, holds a position among the greatest medical societies of the world in many ways unlike that of any other. Born of a felt want by the members of a profession widely scattered in a comparatively new and imperfectly developed country, it struggled along in the weakness of its infancy, conquering the disadvantages of great distances, compassed by comparatively slow, tedious and expensive means of travel, he thought it was not too much to claim that it now stands as the exponent of the medical thought and progress of the profession of the United States.

Although the speaker held that the Association must have its own inherent law, its code of ethics, he believed that the differentiation between so-called schools of medicine should be made alone between ignorance and knowledge. Such differentiation means the abolition of ism and pathy, and there will be introduced in their stead. a more or less accurate interpretation of scientific law as to what constitutes the treatment of disease.

The speaker touched upon the relation of the American Medical Association to the general profession in America, and said that in the general field of medicine, the great rank and file of the profession must continue to work, and as the representative body of this vast army of workers, the American Medical Association, without fear or favor, must continue to be its exponent.

He dwelt upon the relations the Association bore to the State societies, declaring that there was no occasion for jealousy, either between them or the other national societies of our great country; that the field was large and the work ample.

He referred briefly to the evils of intemperance, the matter being one which concerned so closely the members of our own profession, than whom none are better qualified to instruct the public upon the uses and abuses of intoxicating liquors.

As to the question of a National Board of Health, the speaker regarded it as one of great importance; that it had been urged with reason, and he trusted that it would soon be carried into effect. The work of the Department of Agriculture had given great satisfaction, and its field of usefulness was about to be extended by a bill now pending, which has for its purpose the protection of the public against the adulteration of the food products of the United States.

He spoke also of the friendly relations existing between the profession of this and the foreign countries; stating that there were present at the Berlin Congress over six hundred and fifty members from the United States, the largest number representing any nationality except Germany. The speaker then referred to the Pan-American Medical Congress, and said that in the early future the necessity will no longer exist for our advanced pupils making European pilgrimages for original research and study in foreign languages; but rather that our own great centers of learning will be selected as the Meccas of the best modern thought, where students of all nationalities shall gather for instruction.

The address was referred with the thanks of the Association to the committee on publication.

DR. GIHON reported the death of Dr. Brodie, of Detroit, Mich., Ex-President of the Association, and moved a vote of condolence to the family.

DR. BRAY, President of the Canadian Medical Association, was then introduced, and extended a cordial invitation to all members of this Association to attend the meeting of the Canadian Society, at Ottawa, September next.

The report of the Secretary was then read by Dr. Atkinson.

DR. MARSHALL. of Chicago, read the report of the committee on sections, which had been appointed to devise means for improving the work of the various sections The committee advocated that more time be given to section work, by shortening the general sessions. The report was adopted.

A long report of the committee on Secretary of Public Health was then read. After a protracted discussion the report was adopted.

Although there were several committees yet to report, it was so late that an adjournment was had, in order not to encroach upon the time allotted to the work of the sections.

In the evening a reception was tendered to the members of the Association and their ladies at the Light Infantry Armory, by the medical profession of Detroit.

SECOND DAY, WEDNESDAY, JUNE 8TH.

The session was opened at 11 o'clock by the President.

The Secretary read a resolution offered by Dr. Houghton, of Vermont, calling for the appointment of a committee to consider the changes, if any, that it may be deemed necessary to make in the code of ethics. Adopted.

The judicial council reported that the New York State Medical Society and the Erie County Medical Society are not in affiliation with the American Medical Association, and that hence, Dr. W. W. Potter, of Buffalo, as a member of these societies, cannot be recognized as a member of this Association.

Dr. Reynolds, of Kentucky, moved to refer the report back to the council for consideration.

The president decided that the question was out of order. An appeal to this ruling was taken, but the President was sustained.

DR. C. A. L. REED, of the committee on the Pan-American Medical Congress, reported progress.

DR. VANDERVEER, of Albany, here arose to a question of personal privileges as to where he and several others of his colleagues stood, as they were also members of the New York State Medical Society and permanent members of the American Medical Association. After considerable discussion upon the matter and the introduction of several motions, a substitute was finally offered by Dr. King, of Missouri, with an amendment by Dr. Gihon, of the United States Navy, and a further amendment by Dr. Traux to the effect that the President appoint a committee of five to meet a like number from the New York State Medical Society to adjudicate upon the standing of the members who are also members of that society, to report at the next annual meeting and that until such report was made all permanent members from the State of New York now registered, retain their privileges in the Association, which was unanimously carried.

DR. JOHN B. HAMILTON then delivered his address on Surgery, the subject of which was, "The General Principles of the Surgery of the Brain and its Envelopes." The speaker reviewed the history of cerebral surgery from the earliest times down to the present. He said that although the progress in cerebral surgery had been considerable, there yet remained much to gain in this field.

On motion, the address was referred, with the thanks of the Association, to the committee on publication.

After the reading of some notices by the Secretary, Dr. Atkinson, the session was then adjourned.

THIRD DAY, THURSDAY, JUNE 9TH.

DR. GIHON presented the annual report of the Rush Monument Committee.

A resolution was passed endorsing the Association of Medical Colleges in their efforts to raise the standard of professional education.

The report of the trustees of the Journal of the American Medical Association was then presented, and after some discussion, accepted.

The committee on the Jennerial Centennial recommended that the Association devote one day during the meeting in 1896 to eulogies, papers and voluntary essays on Jenner, and the practice of vaccination.

Dr. ALFRED L. GIHON, U. S. Navy, took as his subject of the address on general medicine, "Intellectual Progress in Medicine." He urged an earnest countenance and substantial support of medical literature, both by subscription and contribution.

He said that a minimum of three full courses, in many schools four, and these consequent upon prerequisite academic or collegiate tuition, expressed in one university as six years of continuous collegiate tuition for the intending physician, is a prouder acheivement in medicine in this year, 1892, than all the addition to its pharmacopœia or all the refinements of its therapeutic theories.

The committee on nominations has selected the following candidates:

Dr. Hunter McGuire, of Richmond, Va., President.

Dr. H. O. Walker, of Detroit, first Vice-President.

Dr. H. Brown, of Kentucky, second Vice-President.

Dr. H. Janes, of Vermont, third Vice-President.

Dr. Jesse Hawes, of Greely, Col., fourth Vice-President.

Dr. R. J. Dunglison, of Philadelphia, Treasurer.

Dr. W. B. Atkinson, of Philadelphia, Secretary.

Dr. Montgomery, Assistant Secretary.

Dr. Geo. W. Webster, of Chicago, librarian.

To fill vacancies on the board of trustees: Dr. Alonzo Garcelon, of Lewiston, Maine; Dr. Leartus Connor, of Detroit; Dr. Perry H. Millard, of Minnesota, and Dr. Patterson, of Washington.

Members of the judicial council: Dr. N. S. Davis, of Chicago; Dr. John Morris, of Baltimore; Dr. H. D. Didama, of New York State; Dr. John B. Roberts, of Philadelphia; Dr. A. M. Emmert, of Iowa; Dr W. T. Briggs, of Nashville; Dr. C. W. Voorhes, of Coldwater, Mich.; Dr. W. E. B. Davis, of Rome, Ga.; Dr. A. Morgan Cartledge, of Louisville.

Milwaukee was chosen as the next place of meeting.

### SECTION IN PRACTICE OF MEDICINE.

### FIRST DAY, TUESDAY, JUNE 7TH.

The Relation of Bacteria—Chemical Results to Prophylaxis and Therapeutics, was the title of a paper by Dr. Robert T. Edes, of Jamaica Plain, Mass.

Immunity, by Dr. Kinsman, of Ohio.

Etiology of Specific Disease, by Dr. R. French Stone, of Indianapolis.

Recent Investigations of the Etiology of Diabetes Mellitus, by Dr. S. P. Kramer.

Hepatic Abscess, by Dr. Geo. E. Fell, of Buffalo.

Influenza, by Dr. Jno. E. Link, of Terre Haute.

A Modified Form of Continued Fever following the Epidemic Grippe, by Dr. Jno. H. Hollister, of Chicago.

SECOND DAY, WEDNESDAY, JUNE 8TH.

Positive and Negative Medication, a paper read by Dr. Bedford Brown, of Alexandria, Va.

The Relative Inter-Dependence of Organs in Health and Disease, by Dr. Herrick, of Cleveland, O.

A Case of Amœbic Dysentery, by Dr. E. P. Gerry, of Jamaica Plain, Mass.

Retained Fæces, by Dr. W. D. Christopher, of Chicago.

The Transmission and Behavior of Typhoid Poison as Observed in Country Practice, by Dr. L. N. Davis, of Farmland, Ind.

Catarrhal Gastritis, by Dr. Harold N. Mayer, of Chicago.

The Vertigo of Arterio-Sclerosis, by Dr. Archibald Church, of Chicago.

On Some Non-Valvular Heart Murmurs, by Dr. N. S. Davis, Jr., of Chicago.

The Importance of Position in Examination for Diseases of the Heart, by Dr. O. B. Campbell, of Ovid, Mich.

### SECTION OF SURGERY AND ANATOMY.

FIRST DAY, TUESDAY, JUNE 7TH.

Dr. J. McFadden Gaston, of Atlanta, chairman, read a paper on Surgery of the Gall Bladder and Ducts.

Obstruction of the Cystic Duct, by Dr. W. H. Meyers, of Fort Wayne, Ind.

Peritonitis from Gall Stones, by Dr. W. E. B. Davis, of Rome, Ga.

Gunshot Wound of the Liver and Stomach—Laparotomy—Recovery, by Dr. Jas. T Jelks, of Hot Springs.

Rupture of the Liver, by Dr. Ferger, of Chicago.

Eight Cases of Œsophageal Stricture, by Dr. D. S. Campbell, of Detroit.

SECOND DAY, WEDNESDAY, JUNE 8TH.

The Late Manifestations of Appendicitis and their Treatment, by Dudley P. Allen, of Cleveland.

Desemoid Tumors of the Abdominal Walls, by N. Senn, of Chicago.

Intestinal Lesions in Abdominal and Pelvic Surgery, by Dr. Joseph Price, of Philadelphia.

The Comparative Merits of Inguinal and Lumbar Colotomy, by Dr. J. Matthews.

The Significance of a Hernial Sec, by Dr. E. H. Gregory, of St. Louis.

The Treatment of Injuries of the Abdomen not Requiring Surgical Operations, by Dr. J. Schneck, of Mt. Mormee, Ill.

Emergency Work in Abdominal Surgery, by Dr. Mordecai Price, of Philadelphia.

Bassini's Method for the Cure of Inguinal Hernia applied to a Case Complicated by Undescended Testicle, by Dr. Samuel E. Millikin, of New York.

Hernia, Operable and Inoperable, by Dr. Thos. H. Manley, of New York.

A Few Points on the Management of Strangulated Hernia, by Dr. W. B. Degarmo, of New York.

The Management of Gangrenous Hernia, by Dr. Joseph Ransohoff, of Cincinnati.

The following officers were elected to serve for the ensuing year:

Dr. Jas. T. Jelks, of Hot Springs, Chairman.

Dr. E. T. Tappey, of Detroit, Vice-Chairman.

Dr. Liston Montgomery, of Chicago, Secretary.

